# MUC1 Drives the Progression and Chemoresistance of Clear Cell Renal Carcinomas

**DOI:** 10.3390/cancers16020391

**Published:** 2024-01-17

**Authors:** Emma Bourdon, Thomas Swierczewski, Marine Goujon, Nihad Boukrout, Sandy Fellah, Cynthia Van der Hauwaert, Romain Larrue, Bruno Lefebvre, Isabelle Van Seuningen, Christelle Cauffiez, Nicolas Pottier, Michaël Perrais

**Affiliations:** 1 Univ. Lille, CNRS, Inserm, CHU Lille, Institut Pasteur de Lille, UMR9020-U1277–CANTHER–Cancer Heterogeneity Plasticity and Resistance to Therapies, F-59000 Lille, France; emma.bourdon@univ-lille.fr (E.B.); thomas.sw@hotmail.fr (T.S.); marine.goujon@univ-lille.fr (M.G.); nihad.boukrout@inserm.fr (N.B.); sandy.fellah@univ-lille.fr (S.F.); cynthia.vanderhauwaert@inserm.fr (C.V.d.H.); romain.larrue@univ-lille.fr (R.L.); isabelle.vanseuningen@inserm.fr (I.V.S.); christelle.cauffiez@univ-lille.fr (C.C.); nicolas.pottier@univ-lille.fr (N.P.); 2 CHU Lille, Service de Toxicologie et Génopathies, F-59000 Lille, France; 3 Univ. Lille, CNRS, Inserm, CHU Lille, UMR-S1172, Neuroscience & Cognition, Alzheimer & Tauopathies, F-59000 Lille, France; bruno.lefebvre@inserm.fr

**Keywords:** MUC1, kidney cancer, chemoresistance

## Abstract

**Simple Summary:**

Clear cell renal cell carcinoma (ccRCC) is the main histotype of kidney cancer, which is typically highly resistant to conventional systemic therapies and also to targeted therapies. Identifying the actors and deciphering the molecular mechanisms that lead to tumor progression and/or chemoresistance is an important step in developing new therapeutic strategies to cure ccRCC. In this context, we focused our attention on MUC1, a membrane-bound mucin, which is overexpressed in two-thirds of cancers and known to play a role in tumor progression, chemoresistance, and the establishment of an immunosuppressive microenvironment. In this study, we show that MUC1 overexpression increases the proliferation, invasion, and migration of ccRCC cells and is involved in mediating resistance to conventional and targeted therapies. Overall, our data suggest that MUC1 silencing may represent a potential therapeutic option for ccRCC.

**Abstract:**

While the transmembrane glycoprotein mucin 1 (MUC1) is clustered at the apical borders of normal epithelial cells, with transformation and loss of polarity, MUC1 is found at high levels in the cytosol and is uniformly distributed over the entire surface of carcinoma cells, where it can promote tumor progression and adversely affects the response to therapy. Clear cell renal cell carcinoma (ccRCC), the main histotype of kidney cancer, is typically highly resistant to conventional and targeted therapies for reasons that remain largely unknown. In this context, we investigated whether MUC1 also plays a pivotal role in the cellular and molecular events driving ccRCC progression and chemoresistance. We showed, using loss- and gain-of-function approaches in ccRCC-derived cell lines, that MUC1 not only influences tumor progression but also induces a multi-drug-resistant profile reminiscent of the activation of ABC drug efflux transporters. Overall, our results suggest that targeting MUC1 may represent a novel therapeutic approach to limit ccRCC progression and improve drug sensitivity.

## 1. Introduction

Renal cell carcinoma (RCC) corresponds to 3% of all adult malignant tumors, with clear cell RCC (ccRCC), the main histological subtype, accounting for 70–85% of all RCC cases [1]. ccRCCs at early stages are usually highly resistant to the conventional (cytotoxic) radiation and hormone therapies, and nephrectomy remains, to date, the only curative option. Moreover, 25% of patients present a metastatic disease at diagnosis, and one-third develop metastasis after surgery. The current treatment modalities offered to metastatic patients include the inhibitors of (i) tyrosine kinases (TKIs) and (ii) mTORC signaling, along with (iii) immune checkpoint inhibitors. However, despite their initial response, the majority of metastatic ccRCC patients will develop resistance to these therapies [1,2,3,4]. Therefore, deciphering the molecular mechanisms underlying renal tumor progression and chemoresistance is urgently needed to develop new therapeutic strategies to cure ccRCC.

Mucin 1 (MUC1) is a large type I *O*-glycoprotein translated as a single polypeptide that undergoes autocleavage into N-terminal (MUC1-N) and C-terminal (MUC1-C) subunits, allowing for the formation of a heterodimer through a stable non-covalent association [5]. MUC1-N is an extracellular domain containing a heavily *O*-glycosylated tandem repeat (TR) 20-amino acid (AA) sequence that protrudes far away from the apical side of the cell (200–500 nm). The MUC1-C includes a 58-AA extracellular domain, a 28-AA transmembrane domain, and a 72-AA cytoplasmic tail (CT). In adults, MUC1 expression is cell- and tissue-specific and is altered during carcinogenesis. MUC1 has been shown, in particular, to be overexpressed in two-thirds of cancers and induce cell growth and tumor progression through the activation of various signaling pathways [5,6]. In addition, clinical studies have shown that MUC1 overexpression is associated with chemoresistance in many distinct tumors [7,8]. Mechanistic studies further demonstrated that MUC1 promotes drug resistance by activating the transcription of multi-drug-resistant (MDR) genes [9,10]. For instance, MUC1 induced the resistance of pancreatic cells to gemcitabine and etoposide via the overexpression of four genes belonging to the ABC transporter family: ABCC1, ABCC3, ABCC5, and ABCB1 [9]. Similarly, MUC1 induced the resistance of both lung and cervical cancer cell lines to paclitaxel by upregulating ABCB1 in an EGFR-dependent manner [10]. Furthermore, MUC1 contributes to the self renewal and maintenance of the cancer stem cell (CSC) populations that are known to be involved in mediating resistance to chemotherapy [11].

In ccRCC, MUC1 is known to be diffusely overexpressed, having correlations with tumor progression, nuclear grade, worsened outcome, and metastatic disease [12,13,14,15,16]. MUC1 is also a target gene of the HIF (hypoxia-inducible factor) pathway, which exerts a prominent role in renal carcinogenesis [15]. Furthermore, MUC1 is a driver of the epithelial–mesenchymal transition (EMT) process through the Wnt/β-catenin pathway [17]. MUC1 can also modulate immunoflogosis in the ccRCC microenvironment by activating the classical pathway of the complement system and regulating the immune infiltrate, thereby promoting an immune-silent microenvironment [18]. Finally, tumors expressing high levels of MUC1 are more resistant to cisplatin [16]. Nevertheless, how MUC1 influences the sensitivity of ccRCC to different chemotherapies is poorly documented.

In this study, we showed that the overexpression of MUC1 in ccRCC cells not only promotes multi-drug resistance but also enhances tumor progression.

## 2. Materials and Methods

### 2.1. Cell Lines and Culture Conditions

An ACHN human epithelial renal cancer cell line was purchased from the American Type Culture Collection (ATCC CRL-1611). The RCC4 human mesenchymal-like renal cancer cell line was a gift from Dr. D Bernard (Inserm U1052–CNRS UMR5286, Lyon, France). ACHN cells were cultured with MEM medium (Gibco, Illkirch-Graffenstaden, France) supplemented with 10% of heat-inactivated FBS, 1% of L-glutamine, 0.1% of non-essential amino acids, and 1% of penicillin/streptomycin (Gibco). RCC4 cells were cultured with RPMI 1640 medium (Gibco) supplemented with 10% of heat-inactivated FBS, 1% of L-glutamine, and 1% of penicillin/streptomycin (Gibco). The cells were maintained at 37 °C under a 5% CO_2_ humidified atmosphere. ACHN cells expressing full-length MUC1 are described in [19]. RCC4 MUC1KO cells were obtained through a CRISPR/Cas9 strategy using the “EMA (MUC1) Human Gene Knockout Kit” (Origene, Rockville, MD, USA).

### 2.2. Western Blotting

Total cellular extracts and Western blotting were performed, as previously described [20], using specific primary antibodies (MUC1-C (clone EPR1023, Abcam, Paris, France) and β-actin (clone AC-74, Sigma-Aldrich, Saint-Quentin-Favallier, France)). Peroxidase-conjugated secondary antibodies (A0545 and A9044, Sigma-Aldrich, Saint-Quentin-Favallier, France) were used, and immunoreactive bands were visualized using the West Pico chemiluminescent substrate (Thermo Scientific, Pierce, Illkirch-Graffenstaden, France). Chemo-luminescence was visualized using LAS4000 apparatus (Fujifilm, Courbevoie, France). The original Western blotting data is shown in Supplementary Materials (Figures S1 and S2).

### 2.3. In Vitro Proliferation, Migration, and Invasion Assays

Proliferation and migration were studied using Incucyte technology (Sartorius, Goettingen, Germany). For proliferation, 5000 cells were seeded into each well of a 96-well plate and were placed in the Incucyte enclosure under normal culture conditions. Each well was photographed at 2 locations every two hours. For migration, 15,000 cells were seeded in each well of a 96-well ImageLock plate (Essen Biosciences, Goettingen, Germany) and then incubated under normal culture conditions. Seventy-two hours after seeding, confluent cells were treated for 2 h with 2 μg/mL of mitomycin C (Sigma-Aldrich, Saint-Quentin-Favallier, France), following which a standardized wound was performed thanks to the Woundmaker tool (Essen Biosciences, Goettingen, Germany). Each well was photographed in its center every two hours. For the invasion assays, BD Biocoat Matrigel invasion chambers with 8-μm pores (BD Bioscience, Le Pont de Claix, France) were used. A total of 5 × 10^4^ cells were seeded in the upper chamber in a serum-free medium, while the medium with 10% FBS was added into the lower chamber as a chemoattractant. After incubation for 24 h, the cells that did not invade through the pores were carefully wiped out with a cotton swab. Then, the cells located on the lower surface of the chamber were fixed with the Diff-Quick kit (Medion Diagnostics, Düdingen, Germany), stained with DAPI, and counted.

### 2.4. Cytotoxicity Assay

The cells were seeded onto 96-well plates at a density of 10^4^ cells per well. After 24 h, the medium was replaced with fresh medium containing several drugs (Selleckchem, Souffelweyershein, France): etoposide, oxaliplatin, SN-38, paclitaxel, vinblastine, axintinib, cabozantinib, sorafenib, or sunitinib, at the determined concentration (the range was drug specific) and incubated for 72 h at 37 °C. The viability of the cells was determined using the “Cell titer 96 Aqueous Non Radioactive Cell Proliferation Assay” kit (MTS, Promega, Charbonnières-les-Bains, France), as previously described [21]. At least three independent experiments were performed.

### 2.5. Quantitative RT-PCR

Total RNAs were extracted from the samples with the miRNeasy Mini kit (Qiagen, Courtaboeuf, France), and retrotranscription was carried out on 1 μg of total RNA with the High Capacity cDNA reverse transcription kit (Applied Biosystems, Illkhirch-Graffenstaden, France). The expression levels of ABCC1–6, ABCB1, ABCG2, NANOG, and SOX4 were analyzed using the TaqMan expression assay (Thermo Fisher Scientific, Illkhirch-Graffenstaden, France). The qPCR reaction was performed using the TaqMan Gene expression Master Mix (Applied Biosystem, Illkhirch-Graffenstaden, France), as previously described in [22], using the CFX96 real-time PCR system (Bio-Rad, Steenvoorde, France). The mRNA expression data were normalized to the expression of PPIA (cyclophilin A). The expression levels of mRNA were calculated based on the comparative threshold cycle method (2^−ΔΔCT^) [23].

### 2.6. Efflux Transporter Activity

The activity of efflux transporters was determined using flow cytometry, following the recommendations of the “EFLUXX-ID Gold assay” kit (Enzo Technologies, Villeurbanne, France). The cells were detached through trypsinization and counted. A solution of 1 × 10^6^ cells/mL was prepared and then incubated for 5 min at 37 °C with either specific inhibitors (verapamil, MK-571, and novobiocin) or a medium containing 5% DMSO (control). The eFluxx gold compound was added, and the cells were further incubated for 30 min at 37 °C. Then, 5 μL of propidium iodide were added to assess cell viability. The analysis was performed on the CytoFLEX (Beckman Coulter, Villepinte, France). The multi-drug resistance activity factor (MRAF) was calculated based on the following equation using the mean fluorescence (MFI) of the probe in each condition:

MRAF_Inhibitor_ = ((MFI_Inhibitor_ − MFI_Untreated cells_)/MFI_Inhibitor_) × 100

### 2.7. Statistical Analysis

The data are presented as the mean ± SEM (standard error of the mean). Statistical analyses were performed with GraphPad Prism software version 8 (GraphPad Software, Boston, MA, USA). Statistical differences between each two groups were determined using the Student’s *t*-test. Differences were considered significant when *p* < 0.05.

## 3. Results

### 3.1. MUC1 Increases the Proliferation, Migration, and Invasive Properties of the ccRCC Cell Lines

**Figure 1:**
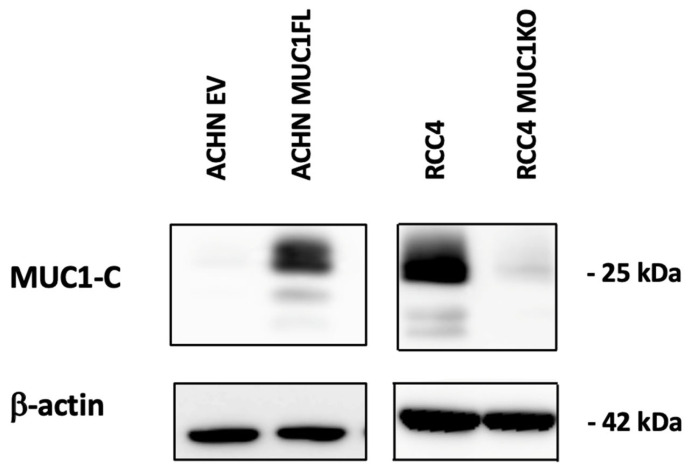
Distinct MUC1 protein abundance in genetically engineered ccRCC cell line models. Whole-cell protein extracts from either MUC1-overexpressing ACHN clones or MUC1-depleted RCC4 clones were analyzed through Western blotting using antibodies against MUC1-C and β-actin.

To assess whether MUC1 influences the tumoral properties of kidney cancer cells, we used renal cancer cell lines that either did (RCC4) or did not express (ACHN) MUC1. We first generated stable ACHN clones expressing full-length MUC1 (MUC1FL) [12], as well as stable RCC4 clones that were engineered to knock down MUC1 expression using CRISPR/Cas9 (MUC1KO) (Figure 1).

**Figure 2:**
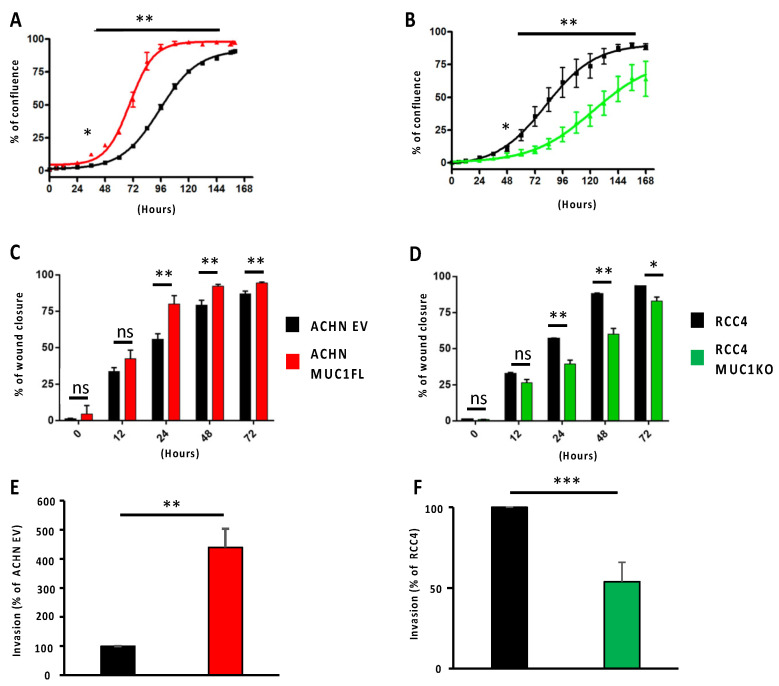
Modulation of MUC1 expression influences the proliferation, migration, and invasiveness of ccRCC cells. Proliferation (**A**,**B**) and migration (**C**,**D**) were assessed using Incucyte technology, whereas cell invasion (**E**,**F**) was evaluated using 24-well Boyden chambers coated with Matrigel^®^ with 10% fetal bovine serum as a chemoattractant. MUC1 overexpression promotes the proliferation, migration, and invasiveness of ACHN cells (**A**,**C**,**E**), whereas MUC1 depletion in RCC4 cells (**B**,**D**,**E**) has the opposite effect. Values are represented as the mean ± SEM and represent at least three separate experiments (* *p* < 0.05, ** *p* < 0.01, and *** *p* < 0.001).

Proliferation was monitored by measuring cell confluence using Incucyte technology. For the ACHN EV cells, a 50% confluence level was observed at around 96 h after seeding, whereas for the ACHN MUC1FL cells, confluence was reached in less than 72 h (Figure 2A; *p* < 0.01). Conversely, the MUC1-deficient RCC4 cell line exhibited a decreased proliferative capacity compared to the control (Figure 2B; *p* < 0.01). Similarly, the wound healing assay showed that the migration of ACHN MUC1FL cells was increased compared to ACHN EV cells (24 h post-wound: 55.8% ± 3.7 for ACHN EV vs. 80% ± 5.6 for ACHN MUC1FL, *p* < 0.01, Figure 2B), whereas the ability of MUC1KO cells to migrate was decreased compared to the control cells (24 h post-wound: 39.4% ± 4.7 vs. 56.8% ± 0.8, *p* < 0.01, Figure 2D). Finally, the invasiveness of ACHN MUC1FL cells was increased compared to ACHN EV cells (4.2-fold, *p* < 0.001; Figure 2E) in contrast to MUC1KO RCC4 cells, which were less invasive (*p* < 0.05; Figure 2F).

Overall, these results demonstrate that MUC1 expression regulates the proliferation, migration, and invasion of renal cancer cells.

### 3.2. MUC1 Increases the Chemoresistance to Conventional Therapies

**Figure 3:**
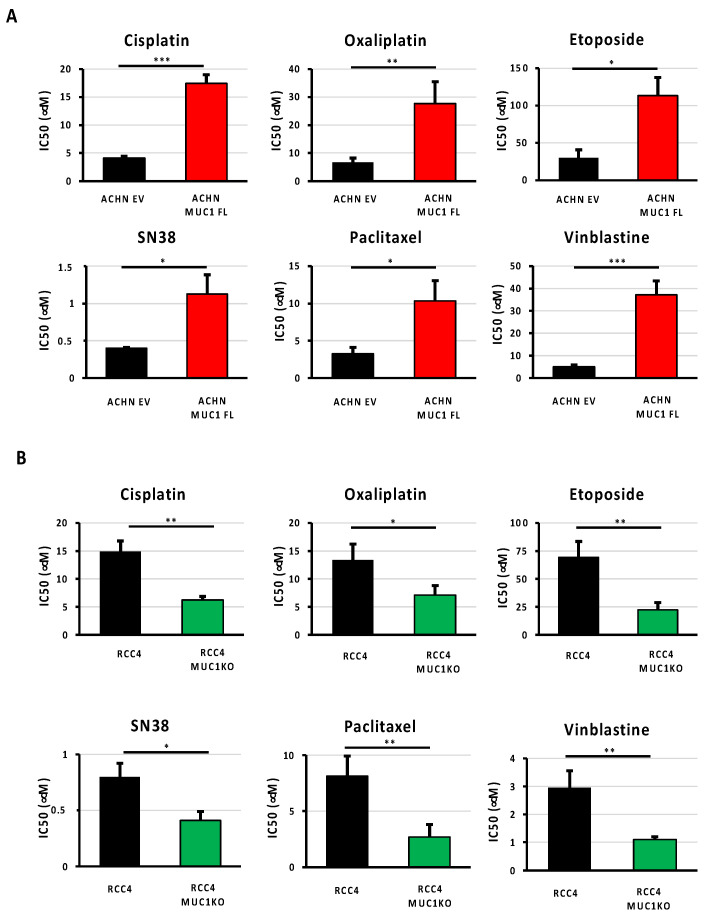
Expression of MUC1 in ccRCC cells is associated with a multi-drug-resistant phenotype. MUC1 ACHN-overexpressing cells (MUC1FL) (**A**), MUC1 RCC4-depeleted cells (MUC1KO) (**B**), and their respective controls were exposed to increasing concentrations of cisplatin, oxaliplatin, etoposide, SN38, paclitaxel, or vinblastine. IC50 (half-maximal inhibitory concentration), a valid marker of drug sensitivity, was determined in each condition after 72 h of drug treatment using the MTS assay. The overexpression of MUC1 confers the ACHN cells a multi-drug-resistant profile (**A**), whereas the depletion of MUC1 in the RCC4 cells (**B**) has the opposite effect. Values are represented as the mean ± SEM and represent at least three separate experiments (* *p* < 0.05, ** *p* < 0.01, and *** *p* < 0.001).

As ccRCCs are usually refractory to conventional chemotherapies, and given that the expression of MUC1 is associated with drug resistance in numerous cancers, such as those affecting the breasts or pancreas [9,10], we investigated whether MUC1 expression also influences renal cancer cell sensitivity to various widely used anti-cancer agents with distinct mechanisms of action. Drug potency was assessed by determining the IC50 (half maximum inhibitory concentration) of the ACHN MUC1FL and RCC4 MUC1KO cells and their respective controls exposed to (i) cisplatin and oxaliplatin, two cytotoxic platinum-based compounds inhibiting DNA replication by forming platinum–DNA adducts; (ii) etoposide, a topoisomerase II poison that promotes cell death by stabilizing topoisomerase 2-induced DNA breaks; (iii) SN38, an active metabolite of irinotecan that exerts anticancer activity by inhibiting DNA topoisomerase I; (iv) paclitaxel, a drug known to inhibit cell division by stabilizing microtubules; and (v) vinblastine, a microtubule-disruptive drug that inhibits mitosis at the metaphase stage through its interaction with tubulin. Our results showed that ACHN MUC1FL cells, which overexpress MUC1, are strongly resistant to all the tested drugs, whereas the drug sensitivity of RCC4 MUC1KO cells, in which MUC1 is knocked out, was markedly and consistently reduced (Figure 3 and Table 1). Altogether, these results unambiguously show that MUC1 overexpression confers a multi-drug-resistant phenotype to ccRCC.

### 3.3. MUC1 Increases the Chemoresistance to Targeted Therapies

**Figure 4:**
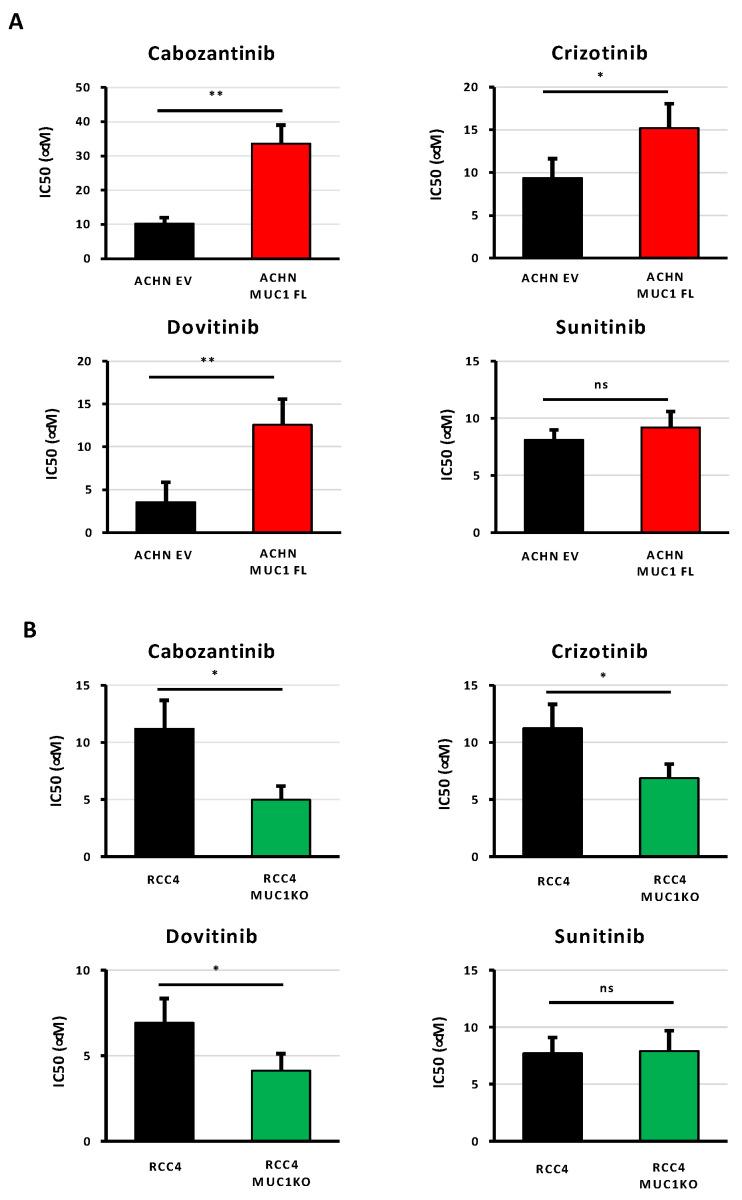
The expression of MUC1 in ccRCC cells is associated with their resistance to targeted therapies. MUC1-overexpressing ACHN cells (MUC1FL) (**A**), MUC1 RCC4-depeleted cells (MUC1KO) (**B**), and their respective controls were exposed to increasing concentrations of cabozantinib, crizotinib, dovitinib, or sunitinib. IC50 (half-maximal inhibitory concentration), a valid marker of drug sensitivity, was determined in each condition after 72 h of drug treatment using the MTS assay. While the modulation of MUC1 has no effect on sunitinib sensitivity (**A**,**B**), its overexpression impaired the responses of ACHN cells to the other tested targeted agents (**A**), whereas its depletion in RCC4 cells (**B**) has the opposite effect. Values are represented as the mean ± SEM and represent at least three separate experiments (* *p* < 0.05 and ** *p* < 0.01).

As ccRCCs are also usually resistant to targeted therapies, we investigated the impact of MUC1 expression on renal cancer cell responses to four multi-targeted receptor TKIs: (i) cabozantinib, an inhibitor of VEGFR, Axl, and Met; (ii) crizotinib, an inhibitor of ALK and c-Met; (iii) dovitinib, an inhibitor of VEGFR, FGFR, and FLT3/c-Kit; and (iv) sunitinib, an inhibitor of VEGFR and PDGFR. The measurements of the IC50 values showed that the modulation of MUC1 expression impacted the sensitivity of ccRCC cells to cabozantinib, crizotinib, and dovitinib, but did not affect the response to sunitinib. In particular, the overexpression of MUC1 enhanced the resistance of ACHN cells to cabozantinib, crizotinib, and dovitinib with an increased IC50 value of 3.3-, 1.62-, and 3.6-fold compared to the control cells, respectively (Figure 4A and Table 2). In contrast, MUC1KO in RCC4 cells exposed to cabozantinib, crizotinib, and dovitinib exhibited a decreased IC50 by 55%, 38%, and 40%, respectively (Figure 4B and Table 2). Overall, these results show that the expression of MUC1 in renal cancer cells is a determinant of targeted therapy responses.

### 3.4. MUC1 Increases the Expression and Activity of Drug Efflux Pumps from the ABC Transporter Family

**Figure 5:**
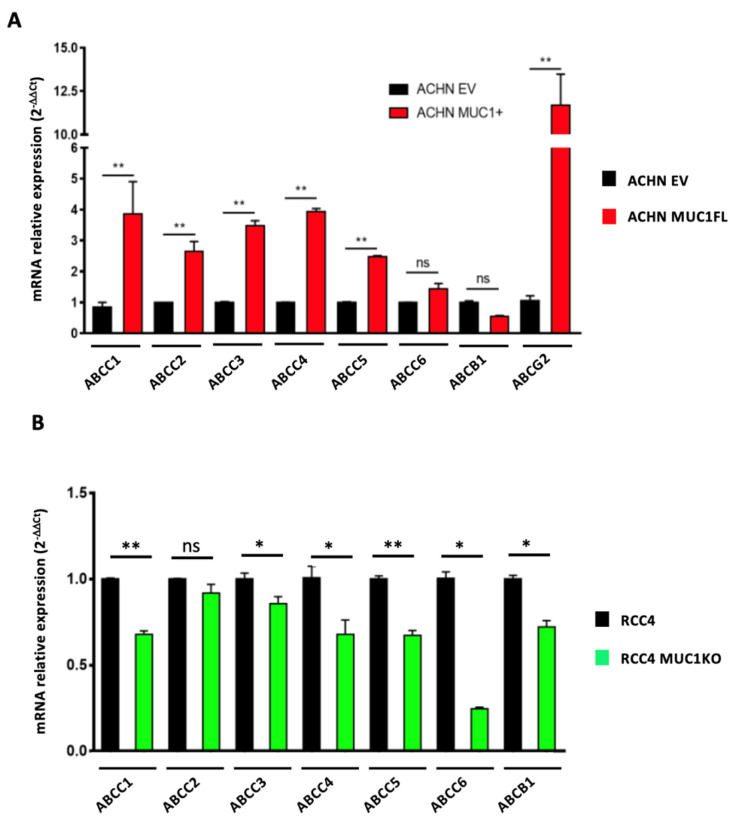
MUC1 increases the expression of numerous efflux pumps. Relative expression of ABCC1–6, ABCB1, and ABCG2 was determined using qPCR in ACHN (**A**) and RCC4 cells (**B**). Overexpression of MUC1 in ACHN cells increases the expression of many members of the ABC transporter family (**A**), whereas its depletion in RCC4 cells (**B**) has the opposite effect. PPIA was used as an internal control. Values are represented as the mean ± SEM and represent at least three separate experiments (* *p* < 0.05 and ** *p* < 0.01).

**Figure 6:**
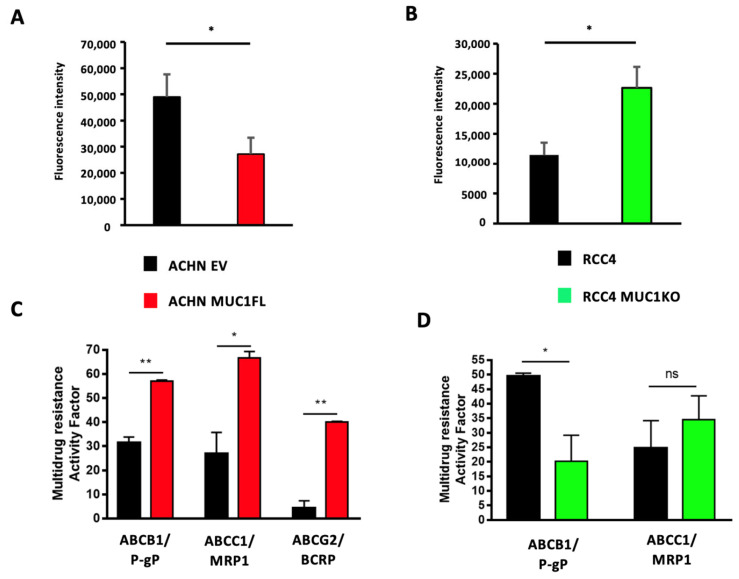
Modulation of MUC1 expression in ccRCC cells influences drug efflux pump activity. The intracellular fluorescence of the eFluxx-ID^TM^ gold detection reagent in ACHN (**A**) and RCC4 (**B**) cells is shown. The multi-drug resistance activity factor (MRAF) represented the P-gP, MDR1, and BCRP activities in ACHN (**C**) and RCC4 (**D**) cells and was determined using the eFluxx-ID^TM^ gold detection reagent in the presence (or not) of verapamil (an ABCB1/P-gP inhibitor), MK-571 (an ABCC1/MRP1 inhibitor), and novobiocin (an ABCG2/BCRP inhibitor). Overexpression of MUC1 in ACHN cells promotes overall drug efflux pump activity (**A**) and ABCB1-, ABCC1-, and ABCG2-specific activity (**C**), whereas its depletion in RCC4 cells decreases the overall drug efflux pump activity (**B**) and ABCB1-specific activity (**D**). Values are represented as the mean ± SEM and represent at least three separate experiments (* *p* < 0.05 and ** *p* < 0.01).

As ATP-binding cassette (ABC) transporters are well known to confer resistance to various cytotoxic and targeted chemotherapies [24], we investigated whether MUC1 expression influences the expression and activity of these proteins by focusing on eight ABC transporters with broad substrate specificity profiles: ABCC1–6, ABCB1 (also known as P-glycoprotein or P-gp), and ABCG2 (or BCRP). For this, we first measured the relative expression of the genes encoding these proteins using RT-qPCR. This showed that ACHN MUC1FL cells exhibit a marked increase in the expression of these transporters with the exception of ABCC6 and ABCB1 (Figure 5A): ABCC1 shows a relative increase in expression of 3.8-fold (*p* < 0.01), ABCC2 2.6-fold (*p* < 0.01), and ABCC3 3.4-fold (*p* < 0.01), whereas RCC4 MUC1KO cells exhibited a reduced expression of these proteins except for ABCC2. RCC4 cells do not express ABCG2. To strengthen these findings, ABC transporter activity was then assessed using the eFluxx ID gold kit (Enzo^®^,Villeurbanne, France). We found that the dye-specific fluorescence was significantly lower in the MUC1-expressing renal cancer cells (ACHN EV = 48 994 ± 8 636 vs. ACHN MUC1FL = 27 178 ± 6259, *p* < 0.05; RCC4 = 11 455 ± 2046 vs. RCC4 MUC1KO = 22 642 ± 3498, *p* < 0.05), suggesting a higher activity of ABC transporters in MUC1-expressing cells, leading to more effective drug effluxes (Figure 6A,B). Chemoresistant MUC1-expressing cells also showed an increase in MRAF (multi-drug resistance activity factor): P-gP, MRP1, and BCRP activities were higher in ACHN MUC1FL compared to ACHN cells (ABCB1/P-gP: 57.1% vs. 31.5%, *p* < 0.01; ABCC1/MRP1: 64.7% vs. 26.9%, *p* < 0.05; and ABCG2/BCRP: 40.1% vs. 4.3%, *p* < 0.01) (Figure 6C). In contrast, in RCC4 cells, compared to RCC4 MUC1KO cells, only P-gP activity was significantly increased (49.5% vs. 20.1%, *p* < 0.05) (Figure 6D). In conclusion, MUC1 increases the expression and activity of efflux pumps from the ABC transporter family.

### 3.5. MUC1 Increases the Expression of Cancer Stem Cell Markers

**Figure 7:**
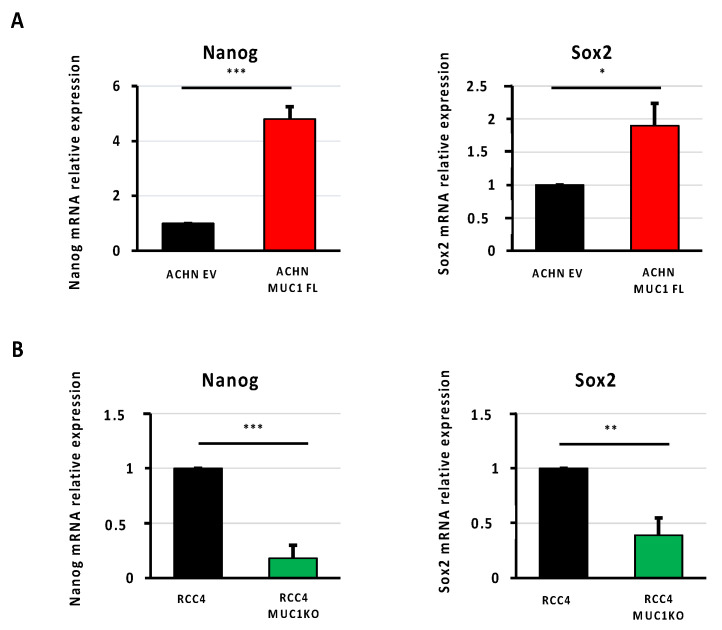
MUC1 increases the expression of cancer stem cell markers. Relative expression of Nanog and Sox2 was determined using qPCR in ACHN (**A**) and RCC4 cells (**B**). Overexpression of MUC1 in ACHN cells increases the expression of the stem cell markers Nanog and Sox2 (**A**), whereas its depletion in RCC4 cells (**B**) has the opposite effect. PPIA was used as an internal control. Values are represented as the mean ± SEM and represent at least three separate experiments (* *p* < 0.05, ** *p* < 0.01, and *** *p* < 0.001).

The cancer stem cell (CSC) phenotype is known to be involved in mediating the resistance to chemotherapy, and MUC1 has been associated with the self renewal and maintenance of CSCs in breast and pancreatic cancers [25,26]. Through RT-qPCR, in our cellular models, we showed that MUC1 expression is associated with an increased expression of two well-known transcription factors involved in stemness: Nanog and Sox2. Their expressions were increased by 4.8- and 1.9-fold in ACHN MUC1FL cells and decreased by 82% and 61% in RCC4 KOMUC1 cells, respectively (Figure 7). In conclusion, MUC1 increases the expression of CSC markers.

## 4. Discussion

Despite the progress achieved over the last decade, drug resistance continues to be the main limiting factor in achieving cures in patients with cancer. Therefore, a better understanding of the molecular and cellular mechanisms driving chemoresistance is of major interest for the development of new effective therapeutic strategies, especially in ccRCC, a common renal carcinoma histological subtype usually refractory to treatments [1,2,3,4]. In this context, we focused on MUC1, a large transmembrane *O*-glycoprotein belonging to the mucin family, overexpressed in approximately two-thirds of solid cancers, including ccRCC [5]. Indeed, previous studies have demonstrated that MUC1 expression is associated with unfavorable outcomes, given its major oncogenic role in tumor progression [8,15,27], chemoresistance [8,9,10,28], and the establishment of an immunosuppressive microenvironment [18,29]. In line with this, we demonstrated in this study, using gain- and loss-of-function approaches, the pivotal role played by MUC1 in ccRCC progression and its response to both conventional and targeted therapies. First, we showed that the overexpression of MUC1 is sufficient to increase ccRCC proliferation, migration, and invasiveness, which are typical cellular features associated with cancer progression. This is consistent with previous studies on breast cancer, showing that MUC1 influences cell proliferation by inducing EGF receptor (EGFR) nuclear translocation and increasing its binding affinity with the cyclin D1 gene promoter [30]. In numerous cancers, such as kidney, breast, and colon cancers, MUC1 also promotes cell migration and invasion by inducing epithelial-to-mesenchymal transition (EMT), a fundamental process by which polarized epithelial cells acquire mesenchymal cell properties with an enhanced motile and invasive capacity [17,31,32,33,34]. Indeed, MUC1 has been notably shown to contribute to the loss of epithelial cell polarity by activating ZEB1, a zinc finger E-box-binding homeobox 1 transcription factor playing a pivotal role in the initiation of the EMT process, through a NF-κB (nuclear factor kappa B) p65-dependent mechanism [31]. Then, we showed that the expression of MUC1 is an important determinant of the ccRCC response to a wide range of anti-cancer drugs with distinct mode of action. In particular, MUC1-overexpressing cells exhibited a multi-drug-resistant profile suggesting the activation of drug efflux transporters, given the unrelated chemical structure of the tested compounds. Indeed, a lot of evidence has suggested that the expression of ATP-binding cassette (ABC) transporters, especially the multi-drug-resistant protein 1 (MDR1, also known as P-glycoprotein or P-gp), which is encoded by ABC subfamily B member 1 (ABCB1), can confer resistance to both cytotoxic and targeted chemotherapies [24]. As expected, our results showed that the modulation of MUC1 strongly influences the expression and activity of ABC transporters, including not only ABCB1 but also other ABC transporters well-described for their implication in drug resistance, such as ABCG2. Nevertheless, whether MUC1 directly or indirectly influences ABC transporter expression in ccRCC remains to be investigated, especially as previous studies have identified MUC1-mediated transcriptional and non-transcriptional mechanisms depending on the tumor type [9,10]. For example, MUC1 has been shown to act as a transcriptional inducer of ABCC1 in pancreatic cancer [9], whereas in cervical cancer, MUC1 promotes ABCB1 expression through an EGFR-dependent mechanism [10]. Finally, as recent reports have shown that MUC1 is expressed in CSCs, and given that these cells typically overexpress ABC transporters, we also investigated whether the modulation of MUC1 may influence the reprogramming of ccRCC and the acquisition of stem cell markers. Interestingly, our results showed that MUC1 expression is closely associated with that of Nanog and Sox2, two established markers of stemness [35]. Altogether, these results suggest that MUC1 may not only aid ccRCC cells to become dedifferentiated and acquire stem cell-like properties but may also promote CSC aggressiveness by upregulating ABC transporters. Furthermore, MUC1 overexpression not only increases the resistance to anoikis, one main feature of CSC, but also circulating tumor cells (CTCs) [19,20,36]. In RCC, MUC1 may represent a new marker of CTCs in combination with other molecules, such as EpCAM and cytokeratins, and thus may improve patient risk stratification and may also be a useful marker to tailor therapy [37]. The immune checkpoint inhibitors (ICIs) used in the clinical treatment management of RCC has revolutionized the therapeutic approach of these tumors, but a significant number of patients remain unresponsive or even develop resistance [38]. A study involving a cohort of 36 ccRCC patients showed that tumors expressing high levels of MUC1 are characterized by an altered metabolism, high M2 tumor-associated macrophage (TAM) response, low immune infiltration, and low expression of PD-L1 [16,18]. MUC1 is also known to be involved in the formation of an immunosuppressive microenvironment since MUC1 (i) increases PD-L1 expression directly at the transcriptional level [29,39,40]; (ii) increases M2 TAMs inside the tumor [41,42]; (iii) decreases MCP-1, IFN-γ, and GM-CSF expression [40]; and (iv) confers cancer cell escape from NK cells [43]. Therefore, targeting MUC1 [44] in ccRCC is of particular interest and warrants further investigations since MUC1 may thus represent a new therapeutic option to eradicate highly drug-resistant CSCs, restore an immunocompetent microenvironment, and sensitize the tumors to ICIs.

## 5. Conclusions

In summary, this study recapitulates the multifaceted oncogenic role exerted by MUC1 in ccRCC and provides additional evidence that targeting MUC1 likely represents a novel therapeutic strategy for refractory cancers.

## Figures and Tables

**Table 1:** The IC50 values of various conventional systemic therapies in ACHN and RCC4 cells.

	Cisplatin (μM)	Oxaliplatin (μM)	Etoposide (μM)	SN38 (μM)	Paclitaxel (μM)	Vinblastine (μM)
ACHN EV	4.1 ± 0.35	6.7 ± 1.56	29.9 ± 11	0.4 ± 0.01	3.25 ± 0.86	5 ± 0.9
ACHN MUC1 FL	17.4 ± 1.54 ***	27.7 ± 7.83 **	113 ± 24 *	1.12 ± 0.26 *	10.35 ± 2.7 *	37.2 ± 6.2 ***
RCC4	14.9 ± 1.87	13.4 ± 2.8	69.2 ± 14.3	0.8 ± 0.12	8.12 ± 1.81	2.96± 0.6
RCC4 MUC1KO	6.22 ± 0.64 **	7.1 ± 1.7 *	22.4 ± 6.4 **	0.41 ± 0.08 *	2.69 ± 1.12 **	1.1 ± 0.1 **

Values are represented as the mean ± SEM. * *p* < 0.05, ** *p* < 0.01, and *** *p* < 0.001 compared to the control cells (ACHN EV vs. ACHN MUC1FL and RCC4 vs. RCC4 MUC1KO).

**Table 2:** The IC50 values of various targeted therapies in ACHN and RCC4 cells.

	Cabozantinib (μM)	Crizotinib (μM)	Dovitinib (μM)	Sunitinib (μM)
ACHN EV	10.21 ± 1.75	9.39 ± 2.24	3.54 ± 2.3	8.1 ± 0.9
ACHN MUC1 FL	33.6 ± 5.4 **	15.2 ± 2.84 *	12.57 ± 3 **	9.2 ± 1.4
RCC4	11.12 ± 2.5	11.24 ± 2.1	6.92 ± 1.42	7.7 ± 1.4
RCC4 MUC1KO	4.98 ± 1.2 *	6.87 ± 1.24 *	4.13 ± 0.99 *	7.9 ± 1.8

Values are represented as the mean ± SEM. * *p* < 0.05 and ** *p* < 0.01compared to the control cells (ACHN EV vs. ACHN MUC1FL and RCC4 vs. RCC4 MUC1KO).

## Data Availability

The data presented in this study are available on request from the corresponding author.

## Supplementary Materials

The following supporting information can be downloaded at: https://www.mdpi.com/article/10.3390/cancers16020391/s1, Figure S1: Whole cell protein extracts from ACHN EV and ACHN MUC1FL were analyzed by western blot using antibodies against MUC1-C and β-actin; Figure S2: Whole cell protein extracts from RCC4 and RCC4 MUC1KO were analyzed by western blot using antibodies against MUC1-C and β-actin.
