# Supplementary material for: MUC1 Drives the Progression and Chemoresistance of Clear Cell Renal Carcinomas
Source: Cancers (Basel). 2024 Jan 17;16(2):391. doi: 10.3390/cancers16020391 (PMC10814283; doi:10.3390/cancers16020391)
Supplement: Supplementary file 1 [file cancers-16-00391-s001.zip › cancers-2798045-supplementary.pdf]

cancers MDPI

Supplementary Materials

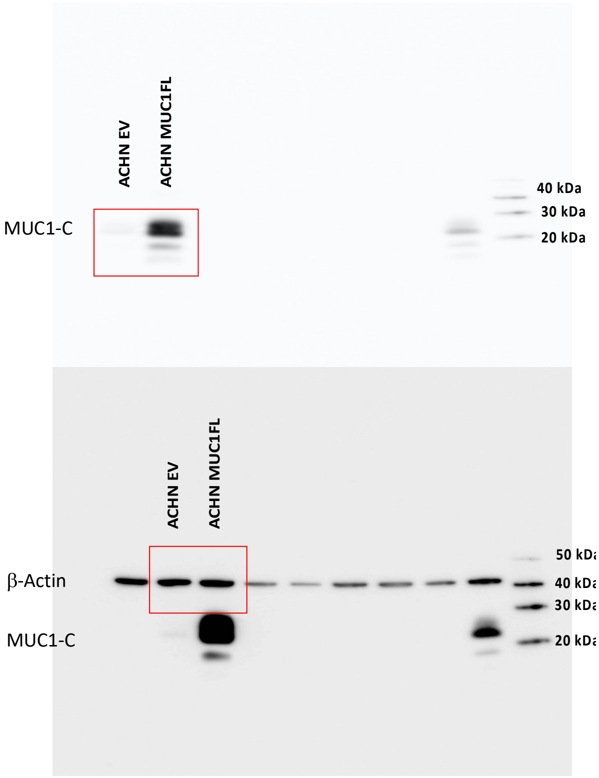

Figure S1. Whole cell protein extracts from ACHN EV and ACHN MUC1FL were analyzed by western blot using antibodies against MUC1-C and β-actin.

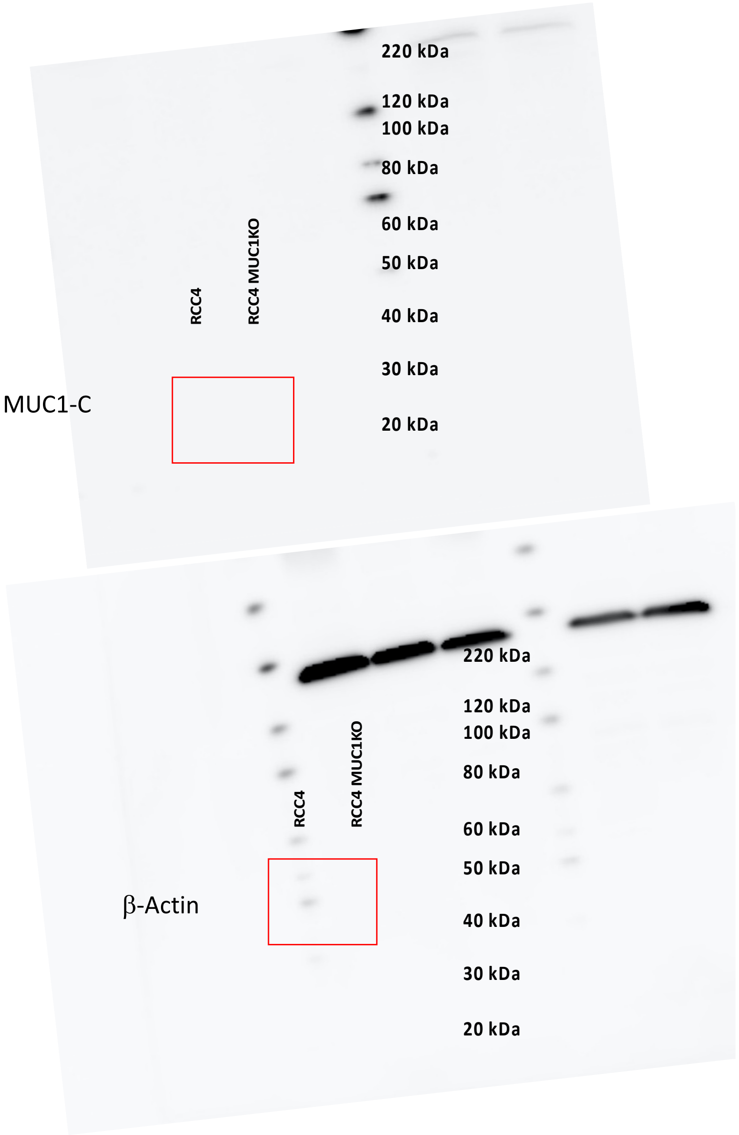

Figure S2. Whole cell protein extracts from RCC4 and RCC4 MUC1KO were analyzed by western blot using antibodies against MUC1-C and β-actin.
